# Lack of increased signal intensity in the dentate nucleus after repeated administration of a macrocyclic contrast agent in multiple sclerosis: An observational study

**DOI:** 10.1097/MD.0000000000004624

**Published:** 2016-09-30

**Authors:** Philipp Eisele, Angelika Alonso, Kristina Szabo, Anne Ebert, Melissa Ong, Stefan O. Schoenberg, Achim Gass

**Affiliations:** a Department of Neurology; b Institute of Clinical Radiology and Nuclear Medicine, Universitätsmedizin Mannheim, University of Heidelberg, Theodor-Kutzer-Ufer 1–3, 68167 Mannheim, Germany.

**Keywords:** dentate nucleus, gadolinium, MRI, multiple sclerosis

## Abstract

Recently, several studies reported increased signal intensity (SI) in the dentate nucleus (DN) after repeated application of gadolinium-based contrast agents (GBCAs), suggesting a deposition of gadolinium in this location. Patients with relapsing–remitting multiple sclerosis (RRMS) frequently show increased permeability of the blood–brain barrier as part of the inflammatory process in the brain parenchyma, which theoretically might increase the risk of gadolinium deposition. In this retrospective study, we investigated a possible increasing SI in the DN after repeated administrations of the macrocyclic contrast agent gadoterate meglumine.

Forty-one RRMS patients (33 women, mean age 38 years) with at least 6 prior gadolinium-enhanced examinations (single dose gadoterate meglumine) were identified. A total of 279 unenhanced T1-weighted examinations were analyzed.

SI ratio differences did not differ between the first and last MRI examination, neither for the DN-to-pons ratio (*P* = 0.594) nor for the DN-to-cerebellum ratio (*P* = 0.847). There was no correlation between the mean DN-to-pons, or between the mean DN-to-cerebellum SI ratio and the number of MRI examinations (*P* = 0.848 and 0.891), disease duration (*P* = 0.676 and 0.985), and expanded disability status scale (EDSS) (*P* = 0.639 and 0.945).

We found no signal increases in the DN after a minimum of 6 injections of the macrocyclic GBCA gadoterate meglumine in RRMS patients. This warrants further investigations in regard to the true pathophysiologic basis of intracerebral gadolinium deposition.

## 1 Introduction

Multiple sclerosis (MS) is a chronic inflammatory demyelinating disease of the central nervous system and the most common cause of nontraumatic neurological disability in young adults. Magnetic resonance imaging (MRI) allows an insight into the pathophysiology of MS and represents currently the most important biomarker for early diagnosis and for disease monitoring. It has therefore been incorporated into the diagnostic criteria by McDonald^[1]^ in 2001 and its importance has been substantiated in the 2005 and 2010^[2]^ revisions. It is well established that MRI disease activity (contrast-enhancing lesions, new or enlarging T2 lesions) is more sensitive than clinical disease activity (relapses).^[3]^ Furthermore, MRI parameters have become important secondary outcome measures in therapeutic clinical trials.^[4]^ Besides, MRI plays an important role in MS-related drug surveillance, for example, in patients treated with natalizumab as a valuable screening method for the detection of progressive multifocal leukoencephalopathy in a presymptomatic early stage.^[5]^

Recently, several studies reported an increased signal intensity (SI) of the dentate nucleus (DN) on unenhanced T1-weighted MRI images after multiple applications of gadolinium-based contrast agents (GBCAs) independent of renal function suggesting a deposition of gadoliniumin this location. Previous studies reported increased SI after multiple doses of linear GBCAs^[6–9]^ suggesting gadolinium deposition, which subsequently has been confirmed in autopsy studies.^[10]^ This raised safety concerns on the use of GBCAs.^[11]^ As a consequence, the European Medicines Agency has recently announced that its Pharmacovigilance Risk Assessment Committee (PRAC) will carry out an in-depth review of the risk of brain deposits and of the overall safety of these products.^[12]^ Recent studies might provide evidence that SI in the DN is associated with the previous administration of linear GBCAs, but possibly not to the same extent with macrocyclic GBCAs.^[13–16]^

In MS, increased permeability of the blood–brain barrier (BBB) is a common finding and a part of the inflammatory process in the brain parenchyma, which might influence the risk of gadolinium deposition. An increase of the permeability of the BBB represents a crucial step in the development of newly developing inflammatory focal brain parenchyma lesions, that are associated with the formation of vasogenic edema and are correlated histopathologically with an inflammatory lymphocytic infiltrate.^[17]^ The opening of the BBB in MS allows GBCAs to cross the BBB and results in the detection of contrast-enhanced lesions.^[18]^ Contrast-enhancement in acute MS lesions varies in shape and size with a mean duration of 3 weeks.^[19]^ Therapeutic intervention by steroid treatment can reduce the extent and shorten the duration of contrast enhancement.^[20]^ Due to these pathophysiological characteristics, MS patients might theoretically pose a higher risk for gadolinium deposition in the brain parenchyma. The aim of this study was to investigate a possible increasing SI in the DN after repeated administrations of GBCAs in MS patients.

## 2 Methods

### 2.1 Patients

From our prospectively collected database, we retrospectively screened patients eligible for further analysis. Inclusion criteria were as follows: diagnosis of definite MS according to the current diagnostic criteria presenting with a relapsing–remitting type,^[2]^ at least 18 years of age, a minimum of 6 gadolinium-enhanced examinations (analogous to the initial study by Kanda et al,^[6]^ who had chosen this cut-off criterion) in our institution on the same MRI system throughout (either 1.5T or 3T) that were all exclusively performed with the macrocyclic contrast agent gadoterate meglumine, and no contrast agent had been previously applied before the first MRI examination. Exclusion criteria were as follows: presence of neurological conditions other than MS (in particular brain hemorrhage, stroke, and tumor); cardiovascular or respiratory disease; current or past substance abuse; current or past radiation or chemotherapy; abnormal renal (estimated glomerular filtration rate <60 mL/min) or liver function (abnormal serum concentrations of aspartate aminotransferase, alanine aminotransferase, total bilirubin, y-glutamyl transpeptidase); lesions located in the dentate nucleus, cerebellum or pons causing difficulties for accurate placement of regions of interest (ROIs); missing or unsatisfactory unenhanced T1-weighted MRI images (e.g., due to motion artifacts); and missing documentation of the applied contrast agent.

The local ethics committee approved this study. Patient consent was not required due to the retrospective nature of the study and the lack of patient interaction.

### 2.2 Magnetic resonance imaging protocol

Initial and follow-up MRI studies were performed on a 3.0-T MR system (MAGNETOM Skyra, Siemens Healthcare GmbH, Erlangen, Germany) or a 1.5-T system (MAGNETOM Sonata, Siemens Healthcare GmbH, Erlangen, Germany).

The standard MRI protocol on the 3.0T system included a high resolution 3D magnetization-prepared rapid acquisition gradient-echo (MPRAGE) sequence (TE = 2.49 ms, TR = 1900 ms, TI = 900 ms, field of view 240 × 240 mm^2^, spatial resolution = 0.9 × 0.9 × 0.9 mm^3^), a 3D fluid-attenuated inversion recovery (FLAIR) data set, proton density (PD) images, T1-weighted images (TE = 2.48 ms, TR = 225 ms, field of view 220 × 220 mm^2^, voxel size 0.7 × 0.7 × 3.0 mm, slice thickness 3 mm) 10 minutes after manual injection of single dose contrast agent (gadoterate meglumine).

On the 1.5T system the protocol included T2-weighted images (slice thickness 3 mm), T1-weighted images (TE = 11 ms, TR = 540 ms, field of view 240 × 240 mm^2^, voxel size = 0.9 × 0.9 × 3.0 mm^3^), FLAIR images, PD-weighted images, followed by T1-weighted images 10 minutes after manual injection of single dose contrast agent, identical to T1-weighted images mentioned previously.

### 2.3 Data processing and analysis

Image post-processing was performed offline on our picture archiving and communication system. Image analysis was conducted on all MRI time points by an experienced reader who was blinded to the serial number of the MRI scan and clinical data. Data analysis was performed in a comparable manner to that reported in previous studies:^[6,16]^ Oval ROIs were placed on the unenhanced T1-weighted images around the left and right DN, the cerebellum next to the DN, and on the central pons. To improve accuracy, we calculated the mean of the left and right DN and cerebellum as proposed previously.^[16]^ If the DN was not clearly visible on unenhanced T1-weighted images, T2-weighted images were used to guide ROI placement on T1-weighted images. First, the average SI of the right and left DN and cerebellum was obtained. Then, the DN-to-pons SI ratio was calculated by dividing the mean SI of the DN by that of the central pons. The DN-to-cerebellum SI ratio was calculated analogue by dividing the mean SI of the DN by the mean SI of the cerebellum.

### 2.4 Statistical analysis

Statistical analysis was performed with IBM SPSS Statistics version 22.0 (IBM Corp., Armonk, NY). A paired *t* test was used to compare the DN-to-pons and DN-to-cerebellum SI ratio between the first and last MRI scans. Correlations between DN-to-pons, DN-to-cerebellum SI, and disease duration and number of MRI examinations were assessed using Pearson correlation coefficient. Correlations between DN-to-pons, DN-to-cerebellum SI and expanded disability status scale (EDSS) were assessed using Spearman rank correlation coefficient.

## 3 Results

A total of 41 MS patients (33 women, 8 men; mean age 38 years [range 18–59 years]) fulfilled the inclusion criteria and were included in the study. The number of contrast-enhanced MRI examinations ranged from 6 to 12 (mean 6.8 examinations, a total of 279 MRI examinations). Mean disease duration was 6.5 years (range 2–16 years) and the median EDSS was 1.5 (range 0–7.5). Thirty-six (88%) patients were on best individually selected treatment either with interferon-beta, glatiramer acetate, fingolimod, natalizumab, or dimethyl fumarate. Twenty patients showed acute contrast-enhancing lesions on post-contrast T1-weighted images (overall 80 contrast-enhancing lesions).

We found no significant differences between the mean DN-to-pons SI ratio of 0.0015 ± 0.0059 (*P* = 0.594) and the mean DN-to-cerebellum SI ratio of 0.007 ± 0.0067 (*P* = 0.847) between the first and last MRI. There was no correlation neither between the mean DN-to-pons nor between the mean DN-to-cerebellum SI ratio and the number of MRI examinations (*P* = 0.848 and 0.891), disease duration (*P* = 0.676 and 0.985), and the EDSS (*P* = 0.639 and 0.945, respectively).

**Figure 1:**
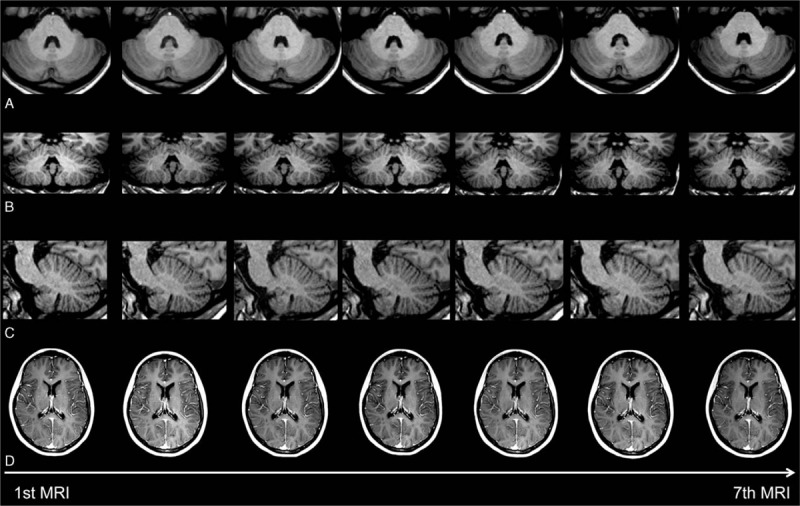
Consecutive (A) axial, (B) coronal, and (C) sagittal unenhanced T1-weighted images of an MS patient at the level of the dentate nucleus. No increase of the signal intensity in the dentate nucleus is observed throughout the examinations. (D) Consecutive MRI examinations of this patient without contrastenhancing lesions.

**Figure 2:**
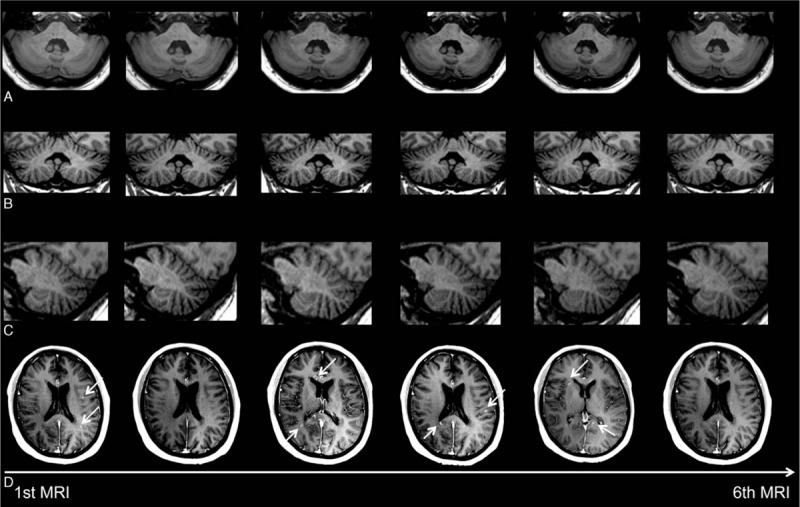
Consecutive (A) axial, (B) coronal, and (C) sagittal unenhanced T1-weighted images of an MS patient at the level of the dentate nucleus. No increase of the signal intensity in the dentate nucleus is observed throughout the examinations. (D) Consecutive MRI examinations of this patient showing multiple contrast-enhancing lesions (white arrows) throughout the 6 examinations.

**Figure 3:**
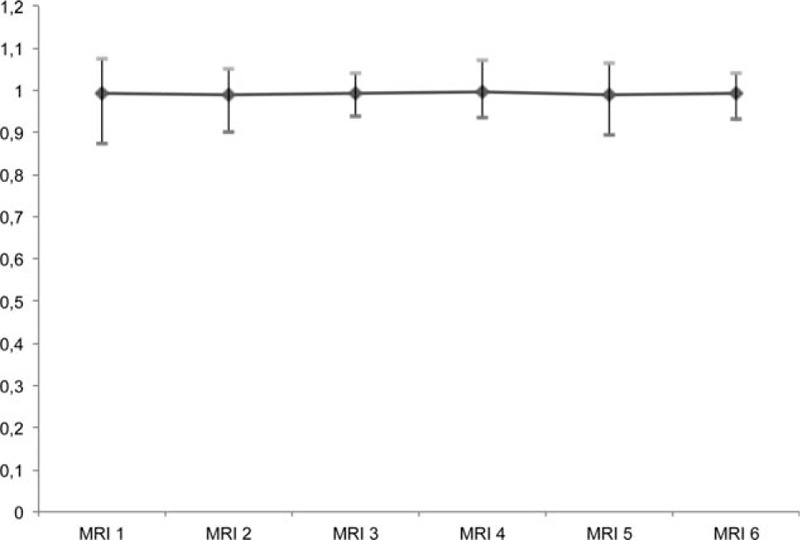
Plot of the dentate nucleus-to-pons signal intensity ratio of 6 MRI examinations in chronological order of all included patients demonstrating stable signal intensities throughout the observation period.

T1-weighted MRI examinations in 2 patients with relapsing–remitting multiple sclerosis (RRMS), at the level of the dentate nucleus, are depicted in Figs. 1 and 2 with 7 and 6 consecutive slices, respectively. Figure 3 shows a plot of the DN-to-pons SI ratio of 6 MRI examinations in chronological order of all included patients.

## 4 Discussion

The present study adds to the growing database of SI changes in the DN after serial applications of GBCAs. In our study, we found no differences of SIs in the DN after multiple administrations of the macrocyclic contrast agent gadoterate meglumine. Our results confirm previous studies that failed to demonstrate an SI increase for the applied macrocyclic GBCAs.^[13–16]^

In patients with MS, in whom repeated contrast-enhanced MRI is routinely performed to diagnose and monitor the disease activity, the experience of abnormal SI in the DN is limited until now. Abnormally hyperintense appearance of the DN on unenhanced T1-weighted images in MS was already described in 2009.^[21]^ These findings were associated with a secondary-progressive disease course, disability, lower normalized brain volume, and higher lesion load, but no association with administration of GBCAs was reported, though patients received either the linear GBCA gadodiamide or the macrocyclic gadoterate meglumine.^[21]^ Errante et al^[7]^ reported an increase of the SI in 38 MS patients after serial application of the linear contrast agent gadodiamide, but no patients receiving macrocyclic GBCAs were included. Recently, a study by Stojanov and colleagues^[22]^ reported for the first time signal abnormalities within the DN after multiple injections of the macrocyclic GBCA gadobuterol in 58 MS patients. However, their study had significant limitations, for example, they could not exclude the use of other contrast agents suggesting a confounding contamination prior to the first MRI examination that was included in the study. In addition, no abnormally hyperintense signal in the DN was visible on the presented unenhanced T1-weighted images.^[23]^

There is another interesting aspect in regard to the findings of increased SI of the DN after serial application of GBCAs. There is increasing evidence that the previously reported signal abnormalities due to T1-shortening after application of contrast agents represent a result of gadolinium deposition in preferred brain areas, as confirmed by animal^[24]^ and autopsy studies,^[10,25]^ although the exact underlying mechanisms are yet unknown. This would be in line with a recent study evaluating effects in T1 and T2∗ relaxometry of the DN in 74 RRMS patients with respect to the number of previous applied GBCAs.^[26]^ The authors found a correlation of increased R1 (=1/T1) values with repeated administration of GBCAs, mainly related to linear contrast agents, while T2∗ relaxometry was not affected, supporting the hypothesis that the observed T1-shortening is related to gadolinium administration and not to iron deposition. In MS, an increase of the permeability of the BBB represents a crucial step in the development of new acute contrast-enhancing lesions. A current study by McDonald and colleagues evaluated autopsy specimens from patients in whom at least 4 MRI examinations with the linear GBCA gadodiamide were performed.^[10]^ Besides the detection of gadolinium clusters in the endothelial walls, they reported that up to 42% of gadolinium had crossed the intact BBB and deposited into the neural tissue interstitium.^[10]^ We found 80 acute contrast-enhancing lesions but no abnormal SIs in the DN after multiple administrations of the macrocyclic contrast agent gadoterate meglumine. One might have assumed that in a disorder that is characterized by repeated focal brain tissue inflammation associated with BBB opening, residuals of gadolinium due to leakage might also be detectable. However, it still remains unclear in what form gadolinium deposition (free ionic form, chelated state) occurs, but the most prevalent hypothesis^[11]^ is that the observed SI changes are a result of dechelation and release of the Gd^3+^ ion from its ligand molecule.^[26]^

After a minimum of 6 applications of the macrocyclic contrast agent gadoterate meglumine, we could not detect gadolinium accumulation in the brain of MS patients. This is in keeping with the results of other recent studies.^[13–16]^ These findings are also motivation to extend our findings to patients with more frequent GBCA applications and longer observations periods.

## Acknowledgment

We acknowledge financial support by Deutsche Forschungsgemeinschaft and Ruprecht-Karls-Universität Heidelberg within the funding programme Open Access Publishing.
